# Relationship between Vitamin D Deficiency and Periodontitis in Korean Adults Aged ≥60 Years: Analysis of Data from the Korea National Health and Nutrition Examination Survey (2013–2014)

**DOI:** 10.3390/ijerph18084181

**Published:** 2021-04-15

**Authors:** Mi-Ra Lee, Su-Jin Han, Hee-Eun Kim, Jun-Seon Choi

**Affiliations:** 1 Department of Dental Hygiene, Hanseo University, Seosan 31962, Korea; leemra1@hanmail.net; 2 Department of Dental Hygiene, College of Health Science, Gachon University, Incheon 21936, Korea; sjhan@gachon.ac.kr (S.-J.H.); hekim@gachon.ac.kr (H.-E.K.)

**Keywords:** oral health behaviors, periodontitis, plasma 25(OH)D, vitamin D, vitamin D deficiency

## Abstract

There have been contradictory reports on the effects of vitamin D in the prevention of periodontitis. We analyzed the association between vitamin D status (levels of plasma 25(OH)D) and periodontitis using the Korea National Health and Nutrition Examination Survey (KNHANES) 2013–2014 database. Among the participants in the KNHANES (2013–2014), only those aged ≥60 years who completed a health interview survey, periodontal examination, and blood test were included in the study. Thus, data from 701 participants were used in the final analysis. Periodontal status was evaluated using the Community Periodontal Index (CPI), and periodontitis was defined as having a CPI score of 3 or 4. Plasma 25(OH)D levels were classified according to two criteria: 20 ng/mL and quartile value. The chi-square test and multivariate logistic regression analyses were performed to analyze the prevalence of periodontitis according to plasma 25(OH)D levels. Univariate analyses showed that periodontitis was not significantly associated with plasma 25(OH)D levels. In the multivariate logistic regression model adjusted for sociodemographic characteristics, the difference in the prevalence of periodontitis between those with a normal range of 25(OH)D and those with low plasma of 25(OH)D levels was not statistically significant. Vitamin D intake has been reported to have benefits in maintaining periodontal health; however, total plasma 25(OH)D levels showed no significant association with periodontitis based on CPI scores in this study. Additionally, these findings reaffirmed the importance of toothbrushing and smoking cessation to prevent periodontitis in people aged ≥60 years.

## 1. Introduction

Periodontitis is a set of inflammatory diseases in which periodontal tissue is destroyed by endotoxins and exotoxins of periodontal pathogens [1]. In particular, *Porphyromonas gingivalis*, a Gram-negative oral anaerobe, is a “keystone” biofilm species that weakens the host’s defense and is involved in the progression of chronic periodontitis [2]. Periodontitis is the main cause of tooth loss in post-middle-aged individuals, for whom it substantially reduces masticatory ability and quality of life [1,3]. Furthermore, several studies have indicated that the presence of periodontal infection and consequent inflammation are associated with increased incidences of atherosclerosis, cardiovascular disease, type 2 diabetes, and rheumatoid arthritis [4,5,6]. 

The primary cause of periodontitis is the persistent accumulation of oral biofilms on tooth surfaces [1], but the progression of the disease is affected by various factors, including host-related factors (e.g., immune disorders and hormonal imbalance) and environmental factors (e.g., smoking and diet) [7,8]. According to a systematic review, it was reported that a large intake of docosahexaenoic acid, vitamins C and E, beta-carotene, milk, fermented dairy products, dietary fiber, fruits, and vegetables contributes to improved periodontal health [9]. Among the many important nutrients in the human diet, vitamin C has attracted attention due to its role in the progression and treatment of periodontitis [1]. Reduced vitamin C levels impede the development and function of leukocytes and directly affect the host immune response [10]. Vitamin C deficiency affects proline hydroxylation, which is involved in collagen maturation and, thus, reduces periodontal tissue regeneration [11,12]. Furthermore, vitamin D can modify the risk of periodontal diseases [13] through the improvement of bone mineral density and other immunomodulatory effects [14]. Vitamin D is first metabolized to 25-hydroxyvitamin D (25(OH)D) in the liver and then converted into 1,25-dihydroxyvitamin (1,25(OH)_2_D), a biologically active form, in the kidneys [15]. A previous study demonstrated that 1,25-dihydroxyvitamin D_3_ (1,25(OH)_2_D_3_), a hormonal form of vitamin D, increases the expression and secretion of antimicrobial proteins, aiding in the treatment of infectious and chronic inflammatory diseases [16]. However, the beneficial effects of vitamin D in maintaining healthy periodontal tissues are controversial [17]. The results of a cross-sectional study suggested that plasma 25(OH)D concentrations were associated with gingival bleeding, an indicator of acute inflammation, but not with chronic periodontitis when evaluated by the alveolar crestal height in combination with tooth loss in postmenopausal women [18]. In a case–control study, participants with low serum levels of 1,25-dihydroxyvitamin tended to be more likely to exhibit periodontitis than participants with higher levels, but this difference was not statistically significant [19].

Periodontal diseases are among the most prevalent diseases worldwide [1] and are the most common cause of visits to medical institutions by Korean adults aged ≥ 65 years [20]. In addition, the prevalence of vitamin D deficiency has recently increased substantially in many countries, including South Korea [21,22,23,24]. Therefore, this study evaluated the relationship between vitamin D status (plasma 25(OH)D levels) and periodontitis in adults aged ≥ 60 years using the Korea National Health and Nutrition Examination Survey (KNHANES) database. 

## 2. Materials and Methods

### 2.1. Participants

We used data from KNHANES VI (2013–2014). Data from the last year of KNHANES VI (2015) were excluded from the analyses because they did not include plasma levels of vitamin D. The KNHANES is a nationally representative survey conducted by the Korea Disease Control and Prevention Agency to investigate the health status, health behaviors, and food and nutrition intake of the Korean population [25]. Among the participants in the KNHANES 2013–2014 (a total of 14,747 people), only those who met the following criteria were included in this study: those aged ≥ 60 years and those who completed a health interview survey, periodontal examination, and blood test. As a result, data from 701 participants were used in the final analysis. Written informed consent was obtained from all participants prior to their inclusion in the KNHANES. KNHANES VI was approved by the Korea Disease Control and Prevention Agency Institutional Review Board (IRB No. 2013–07CON-03–4C and 2013–12EXP-03–5C), and all procedures were performed in accordance with the World Medical Association Declaration of Helsinki. The protocol for this study was approved by the institutional review board of Gachon University (IRB No. 1044396–202101-HR-018–01).

### 2.2. Measurements

This study used data on sociodemographic characteristics (sex, age, household income, and education), oral health behaviors (smoking status, frequency of daily toothbrushing, use of interdental cleaning devices, and dental check-ups in the previous year), systemic health conditions (body mass index and glycated hemoglobin, total cholesterol, high-density lipoprotein cholesterol, low-density lipoprotein cholesterol, triglyceride, and plasma 25(OH)D levels), and periodontal status according to the Community Periodontal Index (CPI). Data on oral health behaviors were categorized as follows: current smoker (yes or no), frequency of daily toothbrushing (≤2 or ≥3 times), use of interdental cleaning devices (yes or no), and dental check-ups in the previous year (yes or no). Body mass index was categorized as underweight (<18.5 kg/m^2^), normal (18.5–24.9 kg/m^2^), and obese (≥25 kg/m^2^) [26]. Glycated hemoglobin levels were categorized as normal (<6.5%) and hyperglycemia (≥6.5%) [27]. The plasma 25(OH)D levels were classified using two criteria. First, when the plasma 25(OH)D level was <20 ng/mL, subjects were considered vitamin D deficient [28]. In addition, plasma 25(OH)D levels were classified into four different groups according to quartile values: lowest (<13.17 ng/mL), middle–low (13.17–17.44 ng/mL), middle–high (17.45–23.40 ng/mL), and highest (≥23.41 ng/mL). All lipids were analyzed using a COBAS 8000 C702 device (Roche, Germany). Total cholesterol and triglyceride levels were determined using enzymatic methods, and high/low-density lipoprotein cholesterol levels were determined using a homogeneous enzymatic colorimetric method [25]. In accordance with the criteria proposed by the National Cholesterol Education Program-Adult Treatment Panel III [29] and the Korean Society for Laboratory Medicine [30], a total cholesterol level of ≥240 mg/dL, high-density lipoprotein cholesterol level of <40 mg/dL, low-density lipoprotein cholesterol level of ≥160 mg/dL, and triglyceride level of ≥200 mg/dL were considered abnormal. Finally, the periodontal status of the participants was evaluated using the CPI [31]. The mouth was divided into sextants: the maxillary arch into three parts (right posterior (#14–18), anterior (#13–23), and left posterior (#24–28)) and the mandibular arch into three parts (right posterior (#44–48), anterior (#33–43), and left posterior (#34–38)). The statuses of gingival bleeding, calculus, and periodontal pockets were evaluated for each index tooth (#16 or #17; #11; #26 or #27; #31; #36 or #37; and #46 or #47) using a CPI probe. Probing was performed by dentists who had received the appropriate calibration training. The CPI score ranged from 0 to 4 as follows: healthy periodontal tissue (CPI 0), gingival bleeding on probing (CPI 1), periodontal tissue with gingival calculus (CPI 2), periodontal tissue with shallow periodontal pockets (4–5 mm) (CPI 3), and periodontal tissue with deep periodontal pockets (≥6 mm) (CPI 4) [25,32]. If there were two index teeth in one sextant, the one with the highest CPI score was chosen as the value for that sextant. In this study, periodontitis was defined as having a CPI score of 3 or 4 [25]. The participants were classified into nonperiodontitis and periodontitis groups.

### 2.3. Statistical Analyses

The collected data were analyzed using the IBM SPSS Statistics software (ver. 23 (IBM Corp., Armonk, NY, USA). Chi-square tests were used to compare the differences in the prevalence of periodontitis according to oral health behaviors and systemic health conditions, including plasma 25(OH)D levels. Additionally, multivariate logistic regression analyses were performed to identify the strength of the association between periodontitis and oral health behaviors or systemic health conditions, including plasma 25(OH)D levels, when sociodemographic characteristics were adjusted for. The multivariate logistic regression model included sociodemographic factors and the factors of which the chi-square test results were *p* < 0.05. The value for determining statistical significance was set at 0.05. 

## 3. Results

### 3.1. Associations between Oral Health Behaviors or Systemic Health Conditions and Periodontitis

The prevalence of periodontitis was higher in participants who were current smokers (63.8%) and who brushed their teeth <3 times per day (52.5%) than in their respective counterparts (*p* < 0.05). Moreover, the prevalence of periodontitis was higher in participants with a glycated hemoglobin value of ≥6.5% (56.7%) than in those with a glycated hemoglobin value of <6.5% (46.3%) (*p* = 0.051). In addition, there was no difference in the prevalence of periodontitis between participants with a plasma 25(OH)D level of <20 ng/mL and those with a level of ≥20 ng/mL. The difference in the prevalence of periodontitis across the plasma 25(OH)D quartiles was also not significant (Table 1).

### 3.2. Strength of Association between Oral Health Behaviors or Systemic Health Conditions and Periodontitis 

In the multivariate logistic regression model adjusted for sociodemographic characteristics, such as age and sex, the participants in the lowest plasma 25(OH)D quartile (<13.17 ng/mL) did not show any difference in periodontitis prevalence when compared with the participants in the highest quartile (≥23.41 ng/mL) (*p* > 0.05). There was no difference in the prevalence of periodontitis between the normal group (plasma 25(OH)D level ≥20 ng/mL) and the vitamin D deficiency group (<20 ng/mL) (*p* = 0.653, data not shown). In addition, participants who were current smokers and who brushed their teeth <3 times per day had more periodontitis than their respective counterparts (*p* < 0.05). Finally, although the participants with a glycated hemoglobin level of ≥6.5% tended to have more periodontitis compared with the participants with a level of <6.5%, this relationship was not significant (*p* = 0.156) (Table 2).

## 4. Discussion

There have been contradictory reports on the effects of vitamin D in the prevention and treatment of infectious and chronic inflammatory diseases, such as periodontitis. Furthermore, both vitamin D deficiency and periodontitis, which substantially affect the quality of life and health of the general population, are increasingly common worldwide [3,21,22,33]. Given these circumstances, further studies on the association between periodontitis and vitamin D deficiency should be conducted using various study designs. In this study, total plasma 25(OH)D levels, the most suitable indicator for the accurate evaluation of systemic vitamin D stores [33], were used to analyze the relationship between vitamin D deficiency and periodontitis (CPI ≥ 3) in Korean adults aged ≥60 years who participated in KNHANES VI (2013–2014). Univariate analyses showed that the prevalence of periodontitis in the participants with vitamin D deficiency (plasma 25(OH)D levels <20 ng/mL) or in the lowest quartile of plasma 25(OH)D levels (<13.17 ng/mL) was not significantly different from that of the respective control group. In the final regression model with adjustment for potential sociodemographic risk factors, total plasma 25(OH)D levels also showed no significant association with periodontitis. Since we analyzed older adults aged ≥60 years, it was difficult to directly compare these results with the findings of previous studies. Vitamin D intake has been reported to have many benefits in maintaining periodontal health due to its direct role in bone metabolism, antibiotic effects against periodontopathogens, and ability to interrupt inflammatory mediators that induce periodontal tissue destruction [34,35]. Despite these important functions of vitamin D, few studies have presented direct evidence that vitamin D status is an important determinant in the development of periodontal diseases [36]. One previous study suggested that men with high vitamin D intake experienced a lower incidence of severe periodontitis and alveolar bone loss [37]. Another study reported that vitamin D can reduce susceptibility to gingival inflammation through its anti-inflammatory effects [38]. Furthermore, one study based on the US National Health and Nutrition Examination Survey database reported that both men and women aged ≥50 years in the lower quintile of serum 25(OH)D had higher periodontal attachment loss than those in the highest quintile [14]. However, in this study, there was no significant association between plasma 25(OH)D levels and attachment loss in patients aged <50 years [14]. In addition, another study suggested that plasma 25 (OH)D levels had more relation to the index measuring acute inflammation, such as gingival bleeding, rather than to the index measuring alveolar bone loss among healthy postmenopausal women [18]. However, contrary to the findings of these previous studies, several additional studies were unable to reveal the beneficial effects of vitamin D on periodontal health. In a clinical study, serum 25(OH)D levels were not associated with periodontal conditions that were assessed, based on gingival bleeding and the presence of periodontal pockets [39]. In addition, even in a study using the KNHANES database like ours, no significant association was found between vitamin D deficiency and periodontal status, except in current smokers [40]. We hypothesized that vitamin D deficiency was not related to periodontitis based on several factors, including the age of the subjects and the plasma 25(OH)D levels. First, the subjects of this study were adults aged ≥60 years, the age group with the highest prevalence of chronic periodontitis in South Korea [25]. Moreover, this age group is more likely to have accumulated more pathogenic oral biofilm, a causative factor of periodontitis, on tooth surfaces than younger age groups [41]. In general, to revert to a healthy periodontal condition, removal of the biofilm must be prioritized using mechanical methods, such as scaling [42]. In other words, the effects of vitamin D on periodontal health may be limited in situations where oral hygiene care is not thoroughly performed. Since there were no data on oral hygiene status in the KNHANES database, this factor could not be considered in this study. Oral hygiene status needs to be controlled for in future studies. Second, vitamin D deficiency has generally been considered as 25(OH)D being <20 ng/mL and insufficiency as 25(OH)D being 21–29 ng/mL without any overt clinical symptoms, even though there has been a lot of controversy over the definition [43]. A review reported that maximum bone mineral density could be achieved when serum 25(OH)D levels reached ≥40 ng/mL, suggesting that vitamin D intake should be higher than the currently recommended amount to obtain a better health outcome [36]. However, it did not precisely define the optimal plasma 25(OH)D concentration. Of the 701 participants in this study, 279 had a serum 25(OH)D level of ≥20 ng/mL, 55 had ≥30 ng/mL, and 7 had ≥40 ng/mL, which shows that as the level increased, the number of people who met the standard decreased significantly. Thus, it was thought that both the plasma 25(OH)D levels of the participants and the thresholds used were too low to analyze the effect of vitamin D on periodontitis. 

In this study, total plasma 25(OH)D levels, a barometer of vitamin D status, were used to analyze the relationship between vitamin D deficiency and periodontitis in Korean adults aged ≥60 years who participated in KNHANES VI (2013–2014). To the best of our knowledge, this study is one of the largest studies to examine the association between vitamin D and periodontitis in older adults. However, this study has several limitations. First, it may not have identified all potential confounding variables. Second, because the KNHANES is a cross-sectional study, it is difficult to identify the causal relationships among the included variables, particularly between periodontitis and plasma 25(OH)D levels. Third, in this study, 20 ng/mL was used as the cut-off value for vitamin D deficiency. However, normal ranges may vary according to age, ethnicity, geographic location, sampling season, and health outcomes [33,44]. Fourth, the CPI has several limitations [45]. In particular, since the CPI score evaluates the periodontal status based on the depth of periodontal pockets using index teeth in each sextant, the extent of inflammation across the dentition may not have been accurately reflected. Finally, because the subjects of our study were limited to those aged ≥60 years, the results cannot be generalized to all age groups. Hence, future studies should elucidate the association between vitamin D status (plasma 25(OH)D levels) and periodontitis using multiple confounding variables and biomarkers that can detect the process of periodontal inflammation more accurately, along with a longitudinal study design.

## 5. Conclusions

The results of analyses of the relationship between vitamin D deficiency and periodontitis based on CPI scores, using the KNHANES database, which represents the health status of the Korean population, indicate that low levels of vitamin D did not appear to be related to the development of periodontitis in adults aged ≥60 years. Additionally, this study reaffirmed the importance of toothbrushing and smoking cessation in preventing periodontitis in people aged ≥60 years. 

## Figures and Tables

**Table 1:** Associations between oral health behaviors or systemic health conditions and periodontitis.

Characteristics	Division		Periodontal Status		χ (*p*)
			Non Periodontitis	Periodontitis	
Oral health behaviors					
Smoking status	Yes	111	43 (36.2)	68 (63.8)	**13.958**
	No	561	305 (55.1)	256 (44.9)	**(0.001)**
Frequency of	<3	403	191 (47.5)	212 (52.5)	**6.975**
daily toothbrushing	≥3	267	155 (57.9)	112 (42.1)	**(0.013)**
Use of interdental	Yes	262	160 (53.1)	132 (46.9)	0.364
cleaning devices	No	379	187 (50.7)	192 (49.3)	(0.628)
Dental check-ups	Yes	190	99 (51.7)	91 (48.3)	0.000
in the previous year	No	477	246 (51.7)	231 (48.3)	(0.995)
Systemic health conditions					
BMI (kg/m^2^)	<18.5	16	11 (72.9)	5 (27.1)	3.935 (0.253)
	18.5–24.9	421	222 (52.5)	199 (47.5)	
	≥25	263	129 (48.6)	134 (51.4)	
Glycated	<6.5	565	302 (53.7)	263 (46.3)	4.972
hemoglobin (%)	≥6.5	135	60 (43.3)	75 (56.7)	(0.051)
TC (mg/dL)	<240	637	329 (51.8)	308 (48.2)	0.113
	≥240	64	34 (49.6)	30 (50.4)	(0.790)
HDL-C (mg/dL)	≥40	551	294 (53.1)	257 (46.9)	2.259
	<40	150	69 (46.4)	81 (53.6)	(0.144)
LDL-C (mg/dL)	<160	116	60 (49.3)	56 (50.7)	0.004
	≥160	9	5 (48.1)	4 (51.9)	(0.942)
TG (mg/dL)	<200	576	298 (52.1)	278 (47.9)	0.364
	≥200	125	65 (49.2)	60 (50.8)	(0.604)
25(OH)D level	<20	422	222 (50.5)	200 (49.5)	0.497
(ng/mL)	≥20	279	141 (53.2)	138 (46.8)	(0.526)
25(OH)D level	<13.17 (lowest)	178	96 (54.0)	82 (46.0)	1.697 (0.694)
(ng/mL)	13.17–17.44	162	85 (48.8)	77 (51.2)	
(quartile)	17.45–23.40	174	86 (49.3)	88 (50.7)	
	23.41 (highest)	187	96 (54.0)	91 (46.0)	
Total		701	363(51.5)	338(48.5)	

BMI, body mass index; TC, total cholesterol; HDL-C, high-density lipoprotein cholesterol; LDL-C, low-density lipoprotein cholesterol; TG, triglyceride. Bold indicates statistical significance at *p* < 0.05.

**Table 2:** Factors associated with periodontitis based on multiple logistic regression analysis.

Factors	Division	Periodontitis	
		OR (CI) *	*p*
Smoking status	Yes (ref. no)	1.758 (1.027–3.008)	**0.040**
Frequency of daily toothbrushing	<3 (ref. ≥ 3)	1.427 (1.018–2.000)	**0.039**
Glycated hemoglobin level (%)	≥6.5 (ref. < 6.5)	1.364 (0.887–2.099)	0.156
25(OH)D level (ng/mL) (quartile)	<13.17 (ref. ≥ 23.41)	0.968 (0.598–1.569)	0.895
	13.17–17.44	1.222 (0.783–1.906)	0.375
	17.45–23.40	1.114 (0.646–1.923)	0.696

* Adjusted for sociodemographic characteristics (age, sex, household income level, and education level). OR, odds ratio; CI, 95% confidence interval. Bold indicates statistical significance at *p* < 0.05.

## Data Availability

The findings of this study were analyzed using a publicly available database. The data are located at: https://knhanes.cdc.go.kr/knhanes/sub03/sub03_02_05.do. (accessed on November 2020).
